# Determinants of Bullying at School Depending on the Type of Community: Ecological Analysis of Secondary Schools in Poland

**DOI:** 10.1007/s12310-017-9206-7

**Published:** 2017-01-11

**Authors:** Joanna Mazur, Izabela Tabak, Dorota Zawadzka

**Affiliations:** 1 Department of Child and Adolescent Health, Institute of Mother and Child, Kasprzaka 17a, 01-211 Warsaw, Poland; 2 Institute of Applied Psychology, The Maria Grzegorzewska University, Szczęśliwicka 40, 02-353 Warsaw, Poland

**Keywords:** Bullying, Ecological analysis, Delinquent behavior, School climate, Urbanization level

## Abstract

Ecological studies, when the school is the unit of analysis, may help to design and evaluate school intervention programs. The paper discusses selected contextual determinants of bullying, using data collected in Poland in 2015 and aggregated to school level (4085 students; 70 junior high schools). The main hypothesis is related to the neighborhood social capital as protective factor and the type of community as a modifier. The main dependent variable was the combined index of bullying which included three perspectives (victim, perpetrator, bystander). Student delinquent behavior was taken into account as potential determinant, along with selected characteristics of the school and neighborhood. The analyses were adjusted for the percentage of the surveyed boys. The overall bullying index ranged, depending on the school, from 0.88 to 4.07 points (out of 12 possible); intraclass coefficient ICC = 2.8%. In the entire sample, the main predictors of bullying were student delinquent behaviors as a risk factor and the school social climate as a protective factor (*R* ^2^ = 56.3%). The stratification of schools due to their location influences the inference regarding those main determinants. The dominating influence of delinquent behavior is visible only in big cities where bullying index showed the highest dispersion. In smaller towns and rural areas, the neighborhood social capital becomes an important protective factor; highly correlated with the school climate. We can conclude that strong social bonds in the community are supportive for school climate and can reduce the level of bullying at schools.

## Introduction

The number of papers about bullying in school environments has increased significantly over the last two decades. Special attention is now given to the phenomenon of bullying, defined as repetitive aggressive behavior toward a student or group of students who are weaker and incapable of defending themselves. This may take the form of physical, verbal, or emotional aggression, with cyber bullying being a new, recent form (Selkie, Fales, & Moreno, 2016). The consequences of bullying experienced in school are usually injuries or trauma, destruction of property, humiliation, social alienation, deterioration in school performance, and even engagement in risk behavior (Fekkes, Pijpers, & Verloove-Vanhorick, 2004; Gini & Pazzoli, 2013; Jiménez-Barbero, Ruiz-Hernández, Llor-Zaragoza, Pérez-García, & Llor-Esteban, 2016; Smalley, Wareen, & Barefoot, 2016; Vaillancourt & McDougall, 2013). The published studies refer to the prevalence of bullying in various populations as well as its determinants (Craig et al., 2009; Elgar, Craig, Boyce, Morgan, & Vella-Zarb, 2009; Harel-Fisch et al., 2011; Molcho et al., 2009). There are an increasing number of systematic reviews concerning the background of bullying and the effectiveness of intervention programs (Chalamandaris & Piette, 2015; Jiménez-Barbero et al., 2016; Park-Higgerson, Perumean-Chaney, Bartolucci, Grimley, & Singh, 2008; Ttofi & Farrington, 2011).

An appropriate assessment of the school environment is of key importance for prevention, as bullying occurs in school. Many studies indicate that the atmosphere in the school is a significant contextual variable affecting experience with bullying (Leadbeater, Sukhawathanakul, Thompson, & Holfeld, 2015; O’Brennan, Bradshaw, & Furlong, 2014; Thapa, Cohen, Guffey, & Higgins-D’Alessandro, 2013). Other factors characterizing the school and the students’ social profile, peer influences, and social status within the peer group may also be important (Espelage, Holt, & Henkel, 2003). Bullying may be an indicator of the overall level of safety in the school, although this is not the only form of violence. The greater the number of students having direct (victims and perpetrators) or indirect (bystanders) contact with bullying, the more dangerous the school. Safety indicators decline in bigger schools which have an unfavorable proportion of students to teachers, in schools located in neglected regions, and in those which have a greater number of students from less affluent families, including families receiving welfare support (Bradshaw, Sawyer, & O’Brennan, 2009). This shows that it is necessary to look at the wider socioeconomic surroundings of the school. The existence of interaction between various individual and contextual factors affecting the threat of bullying in schools has been proven (Gower, McMorris, & Eisenberg, 2015).

An analysis of the prevalence of bullying in Poland and its environmental background (including factors associated with the location of the school) has already been the focus of studies based on the results of major studies of the student population, including successive rounds of Health Behavior in School-aged Children (HBSC) reviews. There is no clear evidence that either an urban or rural environment is more prone to the threat of bullying. In light of data from the newest report describing the results of HBSC 2014 studies, place of residence does not significantly differentiate safety indicators in Polish schools. A considerably higher percentage of perpetrators of bullying has been recorded in the rural community, but mainly among 11-year-olds (that is, in elementary school), and among boys (Malkowska-Szkutnik, 2015). The above analyses have not been adjusted from the point of view of wider characteristics of the neighborhood.

After neighborhood social capital is introduced into the analysis, differences in favor of small towns and villages may be expected, as well as a direct decline in the intensity of bullying and an improvement in the climate of the school. A high level of social capital not only reduces the number of bullies, but also effects a more active and pro-social approach of witnesses who try to help the victims of harassment (Evans & Smokowski, 2015).

Social capital has two basic dimensions: bonding social capital and bridging social capital. The former considerably facilitates cooperation by building a high level of trust in persons who are of a certain community and have shared values. At the same time, however, it makes it more difficult to exchange information with external surroundings. Conversely, bridging social capital promotes encouragement and activates members of various social groups in their joint, inter-group activities (Zajda, 2011). According to well-known Polish social research, “Diagnoza społeczna (The Social Diagnosis)” (Czapinski & Panek, 2015), the highest average social capital indicators are observed in very large cities (above 500 000; *M* = 0.23; SD = 1.15) and tend to decline with the size of the town or village (in villages, *M* = −0.06; SD = 0.09). However, once bonding and bridging social capital indicators are analyzed separately, it is found that only bridging capital increases with the size of the locality, while bonding capital declines (Bednarek-Szczepanska, 2013; Sørensen, 2016).

The dissemination of analyses of the contexts of school bullying is associated with the application of multilevel modeling. Its basis is an analysis of the individual determinants of behaviors associated with factors observed at various levels of the hierarchical data structure. Less empirical work on bullying has focused on the aggregated school data as an object of examination. This approach, which essentially has the character of an ecological analysis, is justified as the preliminary results to multilevel analysis. Focus on the school as the examined unit may provide a great deal of important information which will support programs implemented in schools.

The study involves three issues rarely addressed in the literature: the characteristics of the level of bullying in school not only from the point of view of the perpetrator and victim, but also from that of a witness to the events; emphasis on the difference between schools and not on individual differences and background; the location of schools in areas with different degrees of urbanization is taken into account.

### Objective

The objective of the study is to characterize a selected group of lower secondary schools from the point of view of intensity and environmental background of peer violence defined in literature as “bullying”. Attention was given to the differences associated with the location of the schools according to administrative divisions into urban communities, semi-urban (small cities and suburbs) and rural communities. The study presents hypotheses and answers to research questions concerning relationship between neighborhood social capital and the overall level of bullying at school. The first hypothesis identifies the type of the community as a modifying factor. An assumption was made that the impact of social capital is stronger in smaller communities with better social cohesion than in large cities. The second hypothesis is about interaction of delinquent behavior measured on individual and school level.

The study addresses four research questions. The first two are auxiliary, while the last two directly correspond to above hypotheses.

- To what extent do lower secondary schools differ from each other in terms of the intensity of bullying?
- Which factors characterizing the school environment have the strongest impact on the overall level of bullying in the school?
- Does the overall level of bullying and its background depend upon the size of the town or village in which the school is located?
- Is there an interaction between the selected risk factors for being a bully as measured at the individual level and at the school level?

## Materials and Methods

### Procedure of Data Collection and School Characteristics

The study covered 70 out of 78 lower secondary schools sampled from a total of 234, which underwent a comprehensive external evaluation according to the scheme established for the period of September 2013 to June 2014, as implemented within the framework of the pedagogical supervision system (EES—Education Evaluation System). Data on 4085 students were obtained, of which 48.0% were boys. On average, 58 students per school were examined (SD = 19.7), and the response rate reached 84.8%. The sample is not representative for the whole country as the comprehensive external evaluation is not obligatory and only 3% of schools were on the list. However, on the basis of the obtained material it is still possible to compare better and worse schools, as well as test the selected correlations using the school or student sample.

Table 1 presents the characteristics of the schools by location as given in the EES. No differences were found in terms of gender or age of respondents among the samples of students from schools located in various types of communities. Similarly, the overall assessment of the school standard according to EES was not found to be in any way associated with its location (*F*_ANOVA_(2,67) = 0.704; *p* = 0.498).

**Table 1:** Characteristics of the sample

Variable (source)^a^	Total	Type of community		
		Urban	Semi-urban	Rural
Number of schools	70	28	18	24
Number of students (EES)	286 ± 168	365 ± 186	264 ± 102	209 ± 150
Number of classes (EES)	12 ± 6	14 ± 7	12 ± 4	10 ± 6
School assessment 0–100 (EES)	68 ± 13	70 ± 12	66 ± 15	67 ± 13
Number of surveyed students	4085	1665	1096	1324
Age	15 ± 1	15 ± 1	15 ± 1	15 ± 1
% of boys	48	48	47	50
% of online surveyed	72	85	59	67
% of well-off families	19	24	17	15
Mean final test after VI grade	29 ± 3	30 ± 3	29 ± 2	27 ± 2

*EES* Education Evaluation System; otherwise survey data

^a^M ± SD

The average level of family affluence and final results at the end of elementary school were provided for a given school as additional information. The examination of affluence was based on the FAS scale (*Family Affluence Scale*), applied in HBSC studies and widely described in the literature (Currie et al., 2008). There was an average of 19.2% of students from more affluent families, with significantly more in schools located in urban communities (*χ* ^2^ = 45.36; *df* = 4; *p* < 0.001). According to the information obtained from students, in the studied schools the average result on the final elementary school examination was 28.6 ± 3.0 (out of 40 points). This indicates only a slightly higher result than the national average over the past three years. Urban schools admitted students with significantly higher results on this state examination (*F*_ANOVA_(2,67) = 6.010; *p* = 0.004).

Comparing to the HBSC 2014 data coming from the national school register makes it possible to assess whether our sample is representative. It has been shown that currently described student group is derived from more affluent families and regions of lower level of deprivation than the sample of 4491 of students surveyed within the last HBSC study. The average of family affluence scale (ranged 0–13 points) was 7.25 ± 2.50 and 6.87 ± 2.50, in both samples, respectively (*p* < 0.001). Moreover, the average of the local area well-off scale (ranged 0–6 points) was to 2.87 ± 1.68 and 3.38 ± 1.66, respectively (*p* < 0.001).

Before proceeding with the study, approval was obtained from the Bioethical Commission of the Mother and Child Institute, which evaluated the draft study, the procedure of obtaining parents’ and students’ consent, and the contents of the questionnaire.

It was assumed that the studied sample consisted of lower secondary schools (*N* = 70), not individual students, and the objects of comparison were the indicators obtained from the EES database and data from each student’s questionnaire aggregated to school level.

### Variables and Indicators

#### Outcome Measure

Students answered the questions of how often over the last two months they had participated in bullying another student or students in the school, how often they were the victims of bullying, and how often they had witnessed it. Five categories of answers were given: *did not happen over the past 2* *months, happened once or twice, happened 2–3 times a month, happened about once a week, happened several times a week.* The questions were preceded by an explanation as to what is to be understood as bullying. The concept of this block of questions was based on the report from international HBSC studies; the questions related to being a perpetrator or victim of bullying and the introductory text were identical. A competing classification schema for categorizing students’ bullying involvement was presented by other authors with reference to the standard four-group model: uninvolved, victim, bully, and bully–victim. A new approach in our research is to analyze three participants roles, two described above together with bystanders (Saarento & Salmivalli, 2015). The overall measure of the intensity of bullying in schools was built as a general index which summarized the answers to all three questions. The index has a range of 0–12 points and a reliability level of 0.680.

#### Independent Variables

Apart from school location in urban, semi-urban or rural areas, the following factors *potentially affecting* the bullying index variability, were taken into account: (1) the percentage of boys among respondents at a given school; (2) school size; (3) school quality according to EES; (4) school climate; (5) neighborhood social capital; (6) neighborhood perception; (7) delinquent behavior.

The data from the EES database which was taken into account consisted of the size of the school, in terms of the number of students, and its quality (understood as meeting national standards of education). The collective index of school quality was obtained after analyzing individual evaluation reports (www.npseo.pl). Schools are evaluated by external evaluators on the basis of 12 criteria according to a 5-point scale. A standardized index was calculated with a range of 0–100 points, which may be interpreted as a percentage of the maximum positive score to be obtained.

Three scales derived from the HBSC study report were used for characterizing the schools, with a high score indicating a positive result. The last scale from the Child Health and Illness Profile-Adolescent Edition (CHIP-AE) questionnaire is inversely oriented, whereby a high result indicates a high intensity of negative behavior (Starfield et al., 1995). According to the exploratory factor analysis, all four scales have a single factor structure. Their short descriptions, question examples, and reliability assessment are presented below:

- The scale of SCHOOL CLIMATE with a range of 0–16 points contains four statements concerning support from other students in the class and sense of belonging to the school (e.g., *I feel I belong to my school*); Cronbach’s alpha rate of 0.750;
- The NEIGHBORHOOD SOCIAL CAPITAL scale ranging from 0 to 16 points contains three statements about the level of trust, social bonds, and ability to receive support from neighbors (e.g., *I trust the people who live in the neighborhood*); Cronbach’s alpha rate of 0.771;
- NEIGHBORHOOD PERCEPTION scale ranging from 0 to 6 points contains three statements about physical and social disorder (e.g., *Is it possible to meet groups of problem young people in the area where you live*); Cronbach’s alpha rate of 0.753;
- DELINQUENT BEHAVIORS scale ranging from 0 to 12 points contains three statements concerning aggressive behavior toward other persons or objects, bordering on conflict with the law (e.g., *When was the last time you destroyed something belonging to someone else?*); Cronbach’s alpha rate of 0.828.

In case of the first two scales, answers were given according to five categories (from *certainly agree* to *certainly disagree*). For the third scale, three categories of the intensity of local problems were envisaged (*a lot, not a lot, none*). Five categories of answers were provided for the fourth scale: *never*, *more than a year ago, during the last year, during the last month,* and *during the last week.*

### Statistical Analysis

Statistical analysis used data aggregated to the school level and individual data. As a first step, descriptive statistics were presented, thus illustrating the variability between schools in terms of bullying indicators and their potential background. The correlation rates between the indices described previously were also calculated for the aggregated data. As a next step, schools located in various types of communities were compared. Elements of multilevel analysis were introduced, thereby estimating mixed linear models, separately for different outcome measures. The school identifier was introduced into the models as a random factor and its location as a fixed factor, without any other independent variables. As an element of multilevel analysis, ICCs (*intraclass correlation coefficients*) were also estimated using mixed models procedure, approximating the so-called empty model, with only the school identifier as a random factor. In the next stage of the analysis, classical linear models were estimated on the aggregated data (sample of 70 schools) with an overall bullying index as the dependent variable. A general model was estimated as well as models specific for the three types of communes. A supplementary element of the analysis is the presentation of an example of interaction between factors measured at school and student level, taking being a bullying perpetrator as an outcome. The analysis used the SPSS v.17 statistical package.

## Results

### Distribution of indicators in the lower secondary schools

In our sample, 11.1% of respondents were frequently (2–3 times per month) perpetrators of bullying against other students, while 12.8% were victims of bullying. A much higher percentage (31.0%) declared that they frequently observed such events in their school. At the same time, from the group of witnesses nearly half (47.8%) indicated that they were often perpetrators or victims of bullying. In general, 34.7% of respondents had some kind of frequent contact with bullying (42.3% of boys and 27.8% of girls—*χ* ^2^ = 94.1; *df* = 1; *p* < 0.001). If at least one episode of contact with bullying over the last two months is taken into account, this percentage increases to 61.8% (66.1% of boys and 57.9% of girls—*χ* ^2^ = 28,8; *df* = 1; *p* < 0.001).

The above indicators could easily be compared with the results of HBSC 2014 studies (Malkowska-Szkutnik, 2015). It was found that the percentage of perpetrators of bullying was only 0.3% higher and that of victims was 2.2% higher. After adjustment for differences in the structure of these two samples in terms of gender and age, a statistically significant difference (*p* = 0.004) appears only in the second case.

The overall mean index of bullying was at the level of 2.27 out of 12 possible points. Table 2 presents average values for schools, not weighted by the number of observations. There are high schools in which the scale of bullying exceeds the national average nearly two times, and there are those where this is a trace phenomenon. A similar differentiation between schools was also found with regard to delinquent behaviors.

**Table 2:** Prevalence of bullying and characteristics of social environment in lower secondary schools

	*N*	Total scale range	Distribution of bullying index (*N* = 70 schools)			ICC (%)
			Mean index ± SD	Min–max	K–S (*p*)	
Total bullying index	3937	0–12	2.33 ± 0.66	0.88–4.07	0.794	2.75
School social climate	3973	0–16	9.35 ± 1.01	6.54–12.47	0.990	6.15
Delinquent behaviors	3950	0–12	1.61 ± 0.65	0.55–4.31	0.998	3.22
Neighborhood social capital	3956	0–12	7.49 ± 0.69	6.00–8.86	0.986	4.18
Local area perception	3941	0–6	2.89 ± 0.41	2.16–3.92	0.113	4.06

*ICC* intraclass correlation coefficient estimated by mixed linear model

* Unweighted mean; K–S-level of significance (*p*) in Kolmogorov–Smirnov test for normality

Using the ICC, the school as a local environment was found to have a moderate influence, with the greatest influence being in relation to school climate (6.15%) and the lowest in relation to the index of delinquent behaviors among students (2.75%).

The distribution of the values of the five indices described in Table 2 does not significantly differ from normal distribution in the 70 school sample. For individual data, the normality of distribution was not confirmed in any case.

### Simple Analysis of Relations between Environmental Factors

Table 3 illustrates the correlation rates between the general index of bullying threat in the school and its characteristics, as well as correlation rates between seven other variables describing the sample of lower secondary schools. The general bullying index correlates with five variables—all but school size and quality. This is the only index which significantly correlates with gender. The strongest positive correlation is shown with students’ delinquent behaviors and the strongest negative correlation with positive climate in the school.

**Table 3:** Correlation between scales describing lower secondary schools (*N* = 70)

	1	2	3	4	5	6	7	8
1. Total bullying index		0.000	0.000	0.001	0.040	0.152	0.671	0.025
2. School climate	**−0.547**		0.003	0.000	0.002	0.026	0.672	0.488
3. Delinquent behaviors	**0.639**	**−0.348**		0.000	0.025	0.376	0.849	0.388
4. Neighborhood social capital	**−0.379**	**0.509**	**−0.448**		0.001	0.707	0.086	0.972
5. Local area perception	**−0.246**	**0.368**	**−0.267**	**0.403**		0.236	0.908	0.275
6. School quality index	−0.173	**0.266**	−0.107	0.046	0.143		0.154	0.644
7. School size (students)	−0.052	0.051	−0.023	−0.207	−0.014	0.154		0.120
8. Boys in the sample (%)	**0.268**	−0.084	0.105	0.004	−0.132	−0.056	−0.188	

Correlation coefficient *r* is below and significance level *p* above the diagonal; correlation significant at *p* < 0.05 is bolded

Four basic scales which describe the school environment are also strongly correlated, and the strength of these relations changes depending on school location. It is worth noting how the strength of the relation between school climate and social capital of the neighborhood changes in towns and villages of different size. In urban communities, the obtained correlation rate was *r* = 0.343 (*p* = 0.074), in semi-urban and rural communes, *r* = 0.869 (*p* = 0.000) and in rural communes, *r* = 0.571 (*p* = 0.004). A significant positive correlation between the general neighborhood perception and its social capital appears only in rural communes where *r* = 0.586 (*p* = 0.003).

The quality of the school, as measured by an index from the EES database, has a weaker correlation with other school characteristics. The only significant factor obtained was in relation to school climate.

Table 4 presents a comparison of schools located in various types of communities in terms of the general bullying index and its potential background. Schools located in different types of communities do not differ significantly in terms of their overall level of bullying. However, the dispersion of bullying index is much higher in big cities.

**Table 4:** Prevalence of bullying at school and characteristics of social environment by type of community

Dependent variable	Mean scores by type of community			Fixed effects*	Effect of community type	
	urban	semi-urban	rural		urban	semi-urban
Total bullying index	2.35 ± 0.81	2.33 ± 0.56	2.31 ± 0.55	*F* = 0.022 *p* = 0.979	*t* = 0.208 *p* = 0.836	*t* = 0.092 *p* = 0.927
School social climate	9.33 ± 0.95	9.42 ± 1.17	9.33 ± 0.99	*F* = 0.058 *p* = 0.944	*t* = −0.012 *p* = 0.991	*t* = 0.293 *p* = 0.771
Delinquent behaviors	1.78 ± 0.87	1.36 ± 0.53	1.61 ± 0.72	*F* = 1.742 *p* = 0.183	*t* = 0.830 *p* = 0.410	*t* = −1.068 *p* = 0.289
Neighborhood social capital	7.09 ± 0.60	7.91 ± 0.61	7.66 ± 0.61	***F*** **=** **11.338** ***p*** **<** **0.001**	***t*** **=** **−3.360** ***p*** **=** **0.001**	*t* = 1.345 *p* = 0.183
Local area perception	2.81 ± 0.36	2.95 ± 0.40	2.94 ± 0.48	*F* = 0.940 *p* = 0.396	*t* = −1.160 0.250	*t* = 0.083 *p* = 0.934

Results siginificant at *p* < 0.05 are bolded

* Mixed models—school as random factor; type of localization as fixed factor with rural areas as reference category; *df* = 67 in *F* test; for the effect of community type *t* test statistics with significance level (*p*) is given

Moreover, significant difference was revealed with regard to the general perception of the neighborhood social capital. In that case, the negative differences in large cities are noted with regard to all three component questions. Semi-urban communities are clearly privileged in terms of development of social capital.

### Multivariate Analysis of Bullying Determinants

Table 5 illustrates the results of linear regression analysis estimated in order to identify independent predictors of the level of bullying in high schools. Only significant parameters have been taken into account. In the general model estimated on the basis of mean values for 70 schools, delinquent behaviors of students (as a risk factor) and the school’s social capital index (as a protective factor) proved to be significant predictors of bullying. The third important independent variable was gender. In general, these three factors explain 56.3% of bullying index variability.

**Table 5:** Estimation of multivariate linear regression with total bullying index as dependent variable (adjusted for % of boys in the sample)

Model -independent variables	Unstandardized coefficients		Standardized coefficients	*t*	*p*
	B	SE			
Total—*N* = 70 schools					
Delinquent behavior	0.432	0.076	0.494	5.676	0.000
School social climate	−0.234	0.057	-0.360	−4.140	0.000
*R* ^2^ = 0.563; gender significant at *p* = 0.020					
Schools in urban communities—*N* = 28					
Delinquent behavior	0.798	0.093	0.860	8.603	0.000
*R* ^2^ = 0.740; gender insignificant					
Schools in semi-urban communities—*N* = 18					
Neighborhood social capital	−0.607	0.169	-0.667	−3.585	0.002
*R* ^2^ = 0.445; gender insignificant					
Schools in rural communities—N = 24					
Neighborhood social capital	−0.471	0.138	−0.527	−3.409	0.003
*R* ^2^ = 0.518; gender significant at *p* = 0.001					

After stratifying the examined schools by location, different sets of optimum bullying predictors in schools have been obtained. Delinquent behaviors of students had a significant effect on the school bullying index only in schools located in urban communities. In semi-urban and in rural communes, the neighborhood social capital of the student’s place of residence proved to be an important predictor, its effect not being revealed in the first model. High social capital is an important factor protecting against bullying, and its impact is particularly strong in semi-urban communities. Gender was introduced into the models as a disruptive factor, but it proved significant only in rural locales. Other analyzed factors did not qualify for any model (school quality, school size, general neighborhood perception). Similarly, the effect of family affluence and students’ school results were not proven to be significant.

### Individual and Environmental Risk Factors of Being Bullying Perpetrator

**Fig. 1:**
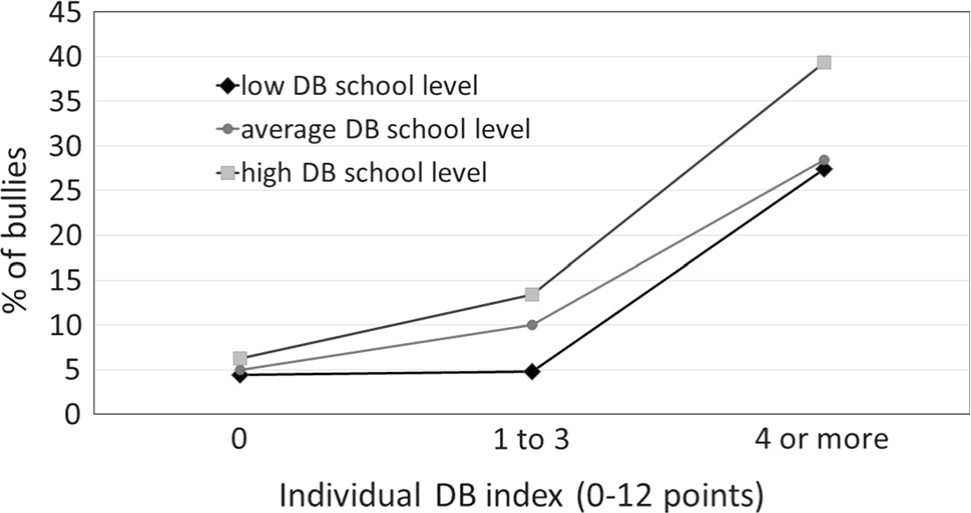
Interaction between delinquent behaviors measured at student and school level as risk factors for repeated episodes of being a perpetrator of bullying in school

Figure 1 illustrates the interaction between delinquent behaviors measured at student and school level as risk factors for repeated episodes of being a perpetrator of bullying in school. The overall percentage of students recently being perpetrators of bullying frequently (2–3 times a month or more) was 11.1%. This percentage shows considerable variability depending upon the students’ inclination to aggressive behavior, and behavior bordering on conflict with the law. Students were divided into three groups depending upon the level of delinquent behaviors’ index: scores of 0, 1–3, and 4 or more points. In these groups, the percentage of perpetrators of bullying was 5.1, 9.5, and 33.2%, respectively. Schools were divided into three equal groups by the average level of the index of these problem behaviors. Only 4.4% of students who do not take up delinquent behaviors and go to schools where this is generally a rare phenomenon confessed to being a frequent perpetrator of bullying. This percentage increases to 39.3% if a person with a high inclination to delinquent behaviors goes to a school where it is a generally accepted norm (Fig. 1).

## Discussion

The study presents data concerning more than 4000 students interviewed in 70 lower secondary schools in Poland at the beginning of 2015. The focus was on comparing schools from the perspective of a general threat of peer violence (bullying). According to the results of HBSC studies, in 2001–2010 in one-third of the 37 countries a declining trend in school bullying was observed. However, bullying remains an important problem for public health, and countries which have managed to reduce the scale of this phenomenon ought to share their experience with others (Chester et al., 2015; Bradshaw, 2015). An equally valuable exchange of information may be achieved within a country by comparing schools with higher and lower bullying level reported by students. The character of the conducted study is an ecological analysis combined with multilevel modeling, and a similar approach was applied in international comparisons (Elgar et al., 2009).

### Outcome Variable

The main variable used to characterize the intensity of bullying in schools was the index, which itself is a lagging indicator of the frequency of being a victim, perpetrator or witness of bullying as reported by students. In general, some kind of contact with bullying over the last two months was acknowledged by 61.8% of respondents, with 44.7% indicating that it was frequent. The alternative scale created on the basis of the study results, without taking witnesses into account, is only half of that. It may also be understated if the young people causing most of the problems do not participate in the survey. The option of being a witness may make it easier for some students to report events in which they may not wish to reveal their own active role. From this point of view including a question about events observed in the school may function as a “vignette” to be used in social studies for collecting data on sensitive subjects.

In light of other study results (Hong & Espelage, 2012), bystanders may either be passive observers of an event (outsiders) or try to help the attacked person (defenders). However, they often clearly side with the perpetrators, encouraging them to continue the attacks or warning them about an approaching adult. Only a few intervention programs include activities aimed at changing the attitudes of students observing bullying in the school (Merrell, Gueldner, Ross, & Isava, 2008; Buckley & Chapman, 2016).

Studies dealing with bullying employ various approaches to define a case. Some researchers adopt a cutoff point of 2–3 instances a month or more. According to Solberg and Olweus (2003), persons who have less frequent contact with bullying only have a disrupted sense of security and are not entirely certain how to classify their experiences. Using the proposed index, it is possible to make use of more information obtained from students and evaluate the level of safety in the school.

### Socio-environmental Background of Bullying

One of the main research hypotheses was related to school location. The study used data aggregated to school level and identified the most important predictors of bullying in school by comparing the general model with models specific to schools located in towns and villages of different size. In the general model and in a big city environment, delinquent behaviors, that is aggressive behaviors bordering on conflict with the law, proved to be an important predictor of bullying. In smaller towns, the protective effect of the neighborhood social capital proved to be dominant. The impact of school climate on the variability of the bullying index is evident only in the general model, while in specific models for various school locations, it is eliminated by the social capital of the neighborhood, which strongly correlates with school climate. Considering that young people spend most of their time outside the home, in school and in their area of residence, these two types of environments which interact with each other should be recognized as having a strong effect on a child’s development (Duncan & Raudenbush, 1999).

Generally, it was demonstrated that schools in cities have more exposure to violence. The results of other research (Harden et al., 2009; Weenink, 2011) lead to similar conclusions. Frequent occurrence of delinquent behaviors among young people in big cities, in comparison with small towns, may be explained by more widespread instances of breaking the law and aggressive behavior, as well as more widespread risk factors leading to such behaviors. Greater differentiation in the level of income of the population which leads to large power imbalances is cited as one of the reasons for the more frequent occurrence of violence in big cities. School children observe and replicate disrupted relations between adults that have resulted from social inequality (Chaux, Molano, & Podlesky, 2009). According to *Social Diagnosis* (Czapinski & Panek, 2015) cited in the introduction, the frequency of experience associated with breaking the law (theft, assault, burglary) in Poland is highest among the inhabitants of big cities. Similarly, according to the European Study of Income and Living Conditions (EU-SILC) in 2013 (Główny Urząd Statystyczny, 2014), the number of households in the country where crime, violence, and vandalism were indicated as social problems was much smaller (2.0%) than that in cities (10.1%).

A research review conducted by Sampson, Morenoff, and Gannon-Rowley (2002) indicates that among the factors determining delinquent behaviors associated with place of residence, poverty and single-parent families play a crucial role, in addition to weak social bonds and low level of social control. The analyses of Stalmach, Tabak, and Radiukiewicz (2014) confirm that in Poland, poverty and being brought up in a single-parent family are important risk factors for young people taking part in bullying (as a perpetrator or victim). Weaker social bonds in big cities compared with small towns also translate into a lower level of bonding social capital as described in the introduction (Bednarek-Szczepanska, 2013; Sørensen, 2016).

Referring to the second hypothesis, a more detailed analysis of the effect of delinquent behaviors on bullying indices leads to interesting conclusions. An accumulation of risk factors measured at the individual and school level was demonstrated. The percentage of perpetrators of bullying increases dramatically among young people taking up delinquent behaviors and going to schools where the percentage of aggressive students is high. Conversely, lower indicators of aggressive behavior in an environment protect students moderately inclined to aggression from becoming a perpetrator of bullying. When writing about the factors which protect young people against violence, Lösel and Farrington (2012) drew attention to the immediate effect and the “buffer” effect. In the latter case, positive action appears in the face of risk, as presented in the study.

### The Strengths and Weaknesses of the Analyses

The fact that the study did not use a representative sample for the entire country may be considered its principal limitation. Only students from schools which had undergone an overall external evaluation during the past two years were included in the study.

Our sample differed from the national HBSC study in terms of the socioeconomic background. The observed difference that interviewed students were from more affluent families and regions should not undermine the reliability of estimated models.

An advantage of the obtained sample, however, is its national scope, territorial differentiation and a significant number of cases examined in a single school, which allowed for an evaluation of the impact of the school environment on the variability of the bullying index. In each school, a similar overall number of students were interviewed at all three levels of teaching, which resulted in an average of one in every five students. The applied multilevel analysis demonstrated that factors associated with the school level explain 2.8% of the variability of the bullying index. This conforms with the results of other research. According to the review published by Azerdo, Levy, Araya, and Menezes (2015) based on the results of thirteen studies, the comparable ICCs ranged from 0.6 to 9.0%, but mainly oscillated around 2–3%. Similarly, the percentages of bullying victims and perpetrators do not dramatically differ from those obtained in parallel national studies carried out on a representative sample of schools (Malkowska-Szkutnik, 2015).

Further limitations of the conducted analysis are caused by the set of variables adopted as potential school bullying predictors. Although consideration of more distant background conditions associated with the exo- and macro-level is an advantage of the study, it lacks a detailed analysis of closer background conditions, including taking into account factors such as the family and relations with important persons.

### Prevention Implications

As indicated by two meta-analyses carried out over the past few years (Ttofi & Farrington, 2009, 2011), anti-bullying programs are capable of reducing violence in schools by approximately 20–23%. Most programs currently employed involve parent training, improved playground supervision, disciplinary methods, classroom methods, and showing videos.

Anti-bullying programs used in Poland and many other countries embrace activities to combat violence in schools on four levels: the school (including cooperation with parents), the classroom, the individual, and the local community, with special emphasis on the first three levels. Taking into account the results cited in the study, the social capital of a neighborhood is to be noted as a factor worthy of consideration when evaluating the above programs because it is capable of modifying their effectiveness. Building social capital requires a wider scope of social intervention and changes in the physical environment, which will support interaction between people and consequently improve social climate and the level of safety in local schools (Eriksson & Emmelin, 2013).

It should also be stressed that the collected empirical material ought to be further analyzed taking into consideration gained results, increasing knowledge about social determinants of being offender, victim or witness of bullying. It is especially important to assess if high social capital of the neighborhood and positive school climate influences different attitudes and behaviors related to bullying similarly.
